# Effect of Citric Acid on Growth, Ecophysiology, Chloroplast Ultrastructure, and Phytoremediation Potential of Jute (*Corchorus capsularis* L.) Seedlings Exposed to Copper Stress

**DOI:** 10.3390/biom10040592

**Published:** 2020-04-11

**Authors:** Aasma Parveen, Muhammad Hamzah Saleem, Muhammad Kamran, Muhammad Zulqurnain Haider, Jen-Tsung Chen, Zaffar Malik, Muhammad Shoaib Rana, Amara Hassan, Ghulam Hur, Muhammad Tariq Javed, Muhammad Azeem

**Affiliations:** 1 Department of Soil Science, University College of Agriculture and Environmental Sciences, The Islamia University of Bahawalpur, Bahawalpur 63100, Pakistan; aasmaparveen452@gmail.com (A.P.); zaffar.malik@iub.edu.pk (Z.M.); 2 MOA Key Laboratory of Crop Ecophysiology and Farming System Core in the Middle Reaches of the Yangtze River, College of Plant Science and Technology, Huazhong Agricultural University, Wuhan 430070, China; saleemhamza312@webmail.hzau.edu.cn (M.H.S.); ghulamhur1993@yahoo.com (G.H.); 3 Key Laboratory of Arable Land Conservation (Middle and Lower Reaches of Yangtze River), Ministry of Agriculture, College of Resources and Environment, Huazhong Agricultural University, Wuhan 430070, China; kamiagrarian763@gmail.com (M.K.); muhammadshoaib1555@gmail.com (M.S.R.); 4 Department of Botany, Government College University, Faisalabad 38000, Pakistan; dr_mzhaider@yahoo.com (M.Z.H.); amarahassangcuf@gamil.com (A.H.); mtariqjaved@gcuf.edu.pk (M.T.J.); 5 Department of Life Sciences, National University of Kaohsiung, Kaohsiung 811, Taiwan

**Keywords:** Jute (*Corchorus capsularis* L.), copper stress, citric acid, antioxidants, plant hormone, chloroplast ultrastructure, phytoremediation

## Abstract

Soil and water contamination from heavy metals and metalloids is one of the most discussed and caused adverse effects on food safety and marketability, crop growth due to phytotoxicity, and environmental health of soil organisms. A hydroponic investigation was executed to evaluate the influence of citric acid (CA) on copper (Cu) phytoextraction potential of jute (*Corchorus capsularis* L.). Three-weeks-old seedlings of *C. capsularis* were exposed to different Cu concentrations (0, 50, and 100 μM) with or without the application of CA (2 mM) in a nutrient growth medium. The results revealed that exposure of various levels of Cu by 50 and 100 μM significantly (*p* < 0.05) reduced plant growth, biomass, chlorophyll contents, gaseous exchange attributes, and damaged ultra-structure of chloroplast in *C. capsularis* seedlings. Furthermore, Cu toxicity also enhanced the production of malondialdehyde (MDA) which indicated the Cu-induced oxidative damage in the leaves of *C. capsularis* seedlings. Increasing the level of Cu in the nutrient solution significantly increased Cu uptake by the roots and shoots of *C. capsularis* seedlings. The application of CA into the nutrient medium significantly alleviated Cu phytotoxicity effects on *C. capsularis* seedlings as seen by plant growth and biomass, chlorophyll contents, gaseous exchange attributes, and ultra-structure of chloroplast. Moreover, CA supplementation also alleviated Cu-induced oxidative stress by reducing the contents of MDA. In addition, application of CA is helpful in increasing phytoremediation potential of the plant by increasing Cu concentration in the roots and shoots of the plants which is manifested by increasing the values of bioaccumulation (BAF) and translocation factors (TF) also. These observations depicted that application of CA could be a useful approach to assist Cu phytoextraction and stress tolerance against Cu in *C. capsularis* seedlings grown in Cu contaminated sites.

## 1. Introduction

Copper (Cu) has been recognized an essential micronutrient required for normal growth and development by all living organisms [1,2,3]. Moreover, Cu plays a significant role in many physiological, biochemical and metabolic processes such as oxidation-reduction reactions, protein synthesis, oxygen carrier (hemocyanin) synthesis, carbohydrate, protein, and cell wall metabolism, nitrate reductase, and nitrogen fixation mechanisms [4,5,6]. However, Cu in cells need to be kept at low levels because excessive Cu can alter DNA structure, membrane integrity, photosynthesis, alterations in chloroplast structure and respiration which affect plant growth and development [7,8,9,10]. Distribution of Cu in soil is impacted by climatic, geological, and pedological factors. In addition to geological sources and industrial pollution, other anthropogenic sources related to the agricultural practices, for example, repeated use of Cu containing agrochemicals, may increase Cu levels in soils [1]. In China, more than 16.1% soils are contaminated with different heavy metals including 2.1% Cu contaminated soils [11]. Furthermore, Cu is present in the form of complexes or particulate matter in different fresh water bodies such as lakes, ponds, and rivers [1]. The Scottish Pollutant Release Inventory (SPRI) emission reporting threshold for Cu and Cu compounds is 20 kg per year pollutant emission to water. When Cu releases into water, the dissolved Cu can be carried in surface waters either in the form of free Cu or its compounds and eventually deposits in the sediments of rivers, lakes, and estuaries or bound to particles suspended in the water. Although Cu binds strongly to suspended particles and sediments, there is evidence suggesting that some of the water-soluble Cu compounds do enter ground water [1]. The cell membranes of plants are considered as primary sites of injury due to heavy metals and membrane destabilization was frequently attributed to lipid peroxidation [12,13]. Large number of active oxygen free radicals in plant tissues under stress will cause cell membrane lipid peroxidation, which will damage normal structure and function of membrane [14,15]. Malondialdehyde (MDA) is an oxidized product of membrane lipids, which is commonly considered general indicator of lipid peroxidation as well as stress level [16,17]. The antioxidative defense system in plants plays an important role in reducing toxicity of heavy metals [5,15,18,19,20]. With excessive Cu stresses, the induction of antioxidative enzymes, including superoxide dismutase (SOD) and peroxidase (POD) play an important role in mechanism of reducing Cu toxicity in plants, and plants may secrete some substances to reduce toxicity of Cu for example in rice, ramie, and maize [21,22,23].

Environmental bio-technology is a new discipline which integrates living materials, mainly plants, and very small animals like earth worms, microorganisms to address the problems of environmental management and sustainable development [24,25]. Phytoremediation, a green technology which uses specific plant species to rehabilitate soil contaminated with metals and other harmful materials and is considered to be a cost effective, reliable, eco-friendly, and scientifically approved method [26,27,28]. This concept was first proposed by Chaney [29] and then developed through the study of plant species ability to remove pollutants from environment components. It can be used for a wide range of organic and inorganic contaminants [11,28,30]. In this regard, phytoextraction is a type of phytoremediation in which the plant absorbs heavy metals from the soil or water and transports it to different harvestable parts of the plant [26,31]. Various hyperaccumulator species have been used previously for the phytoextraction of different toxic metals such as zinc (Zn), mercury (Hg), lead (Pb), and cadmium (Cd) [32,33,34,35]. Recently, many fibrous crops have also been used for the phytoextraction of heavy metal [5,16,21]. Among different fibrous crops, jute (*Corchorus capsularis* L.) has been considered more tolerant to heavy metals stress due to its specific physiological and biochemical activities [36]. Moreover, its huge biomass production and deep rooting system makes it a potential candidate for the heavy metal stress environment [3,18]. In our previous studies, we have concluded that *C. capsularis* is a hyperaccumulator species for the Cu-contaminated soil [3,18,36,37]. The major characteristics of *C. capsularis* which make it an excellent candidate for phytoremediation of heavy metals and post-harvest advantages of raw-jute has been discussed in detail in our review of literature on *C. capsularis* [31].

Success of phytoextraction depends upon the metal solubility and availability in soil for root uptake. Moreover, metal bioavailability of the plant mainly depends upon the physio-chemical properties of the soil such as soil pH, cation exchange capacity and electrical conductivity [8,38,39]. Usually, many of the heavy metals are adsorbed in soil particles to make soil aggregates that are hard to be integrated by plants. Thus, the use of acids, which are low molecular weight organic acids like citric acid (CA), is crucial to alter the chemical activity/bioavailability of heavy metals and improve phytoextraction [30,40,41]. For enhancing phytoextraction, CA is the commonly used synthetic chelator. However, its slow degradation rate and long persistence in soil makes it unsuitable for beneficial purpose. Many studies documented the chelating potential and plant growth promoting role of CA under different heavy metals such as Cu [30], Cd [41], Pb [42], and Cr [39]. There are many previous studies on different heavy metals using CA as the chelators in many different plant species [40,41,43], but very few literatures are available on the phytoextraction of Cu using *C. capsularis* as a hyperaccumulator species. Therefore, the evaluation of morphological traits, ecophysiological responses, and phytoextraction potential of *C. capsularis* under high levels of Cu contamination with or without the application of CA is required. The results from this study will add to our knowledge about the effects of exogenous CA application (i) on growth, physiological responses, and alterations in cellular organelles of *C. capsularis* under Cu stress and (ii) Cu uptake and transport to different parts of *C. capsularis* and detoxification of Cu toxicity under the application of CA when grown in highly Cu contaminated sites.

## 2. Materials and Methods

### 2.1. Plant Growth Conditions and Treatments

The seeds of jute (*Corchorus capsularis* L.) used in the current study were collected from Bast and Fiber Research Center, Huazhong Agricultural University, Hubei Province, P.R. China. The seed of C-3 plant (*C. capsularis*), which is a type of white jute and originated from Bangladesh were subjected to sterilization using 1% (*w*/*v*) sodium hypochlorite for 15 min followed by washing with distilled water for the prevention of surface fungal/bacterial contamination. The seeds were sow in the experimental station of Huazhong Agricultural University Wuhan, Hubei, China (114.20′ E, 30.28′ N; 50 m above sea level). After two weeks of seed sowing uniform sized seedlings were transferred to a volumetric flask (150 mL) containing Hoagland nutrient solution (pH 6.2). The composition of Hoagland’s nutrient supplied to *C. capsularis* seedlings was as follows (μmol L^−1^): Ca(NO_3_)_2_, 2000; KH_2_PO_4_, 100; KNO_3_, 3000; MgSO_4_, 1000; H_3_BO_3_, 50; MnCl_2_·4H_2_O, 0.05; ZnSO_4_·7H_2_O, 0.8; CuSO_4_·5H_2_O, 0.3; H_2_MO_4_·H_2_O, 0.10; and FeNa-CA, 12.5. The volumetric flasks were placed in the growth chamber (day/night temperature at 25/20 °C) with 12 h light (13,000 lx) and 12 h dark (HP250GS-C, Ruihua Instrument and Equipment Co., Ltd., Wuhan, Hubei, China) of Huazhong Agricultural University. Plants were able to grow in nutrient solution and after three days of plants transferred, nutrient solution was spiked artificially with various levels of Cu i.e., (0, 50, and 100 μM) using CuSO_4_. 5H_2_O (99% purity). Citric acid (CA) (2 mM) was also added having Cu concentrations and experiment was executed in complete randomized design (CRD) having one plant in each flask with six replications of each treatment. Following treatment plan was executed: (1) Cu_0_CA_0_ (Cu = 0 μM and CA = 0 mM); (2) Cu_0_CA_1_ (Cu = 0 μM and CA = 2 mM); (3) Cu_1_CA_0_ (Cu = 50 μM and CA = 0 mM); (4) Cu_1_CA_1_ (Cu = 50 μM and CA = 2 mM); (5) Cu_2_CA_0_ (Cu = 100 μM and CA = 0 mM); (6) Cu_2_CA_1_ (Cu = 100 μM and CA = 2 mM). We used 2 mM of CA and 50 and 100 μM Cu in the nutrient solution as these concentrations of Cu but slightly higher concentration of CA (2.5 mM) have already been used in the literature such as Zaheer et al. [30]. The nutrient solution along with Cu and CA levels was renewed thrice in a week to protect from any microbial or fungal attack replaced Cu and CA solution and also no symptoms of waterlogging were visible in the time span of the experiment. The pH of nutrient solution was maintained (6.2 ± 0.2) throughout the experiment using 1M H_2_SO_4_ and NaOH. After three weeks, all plants were wrapped for different morphological traits, gaseous exchange parameters, antioxidants, and metal accumulation in different parts of plant on April 2019. 

### 2.2. Sampling and Data Collection

After 21 days of experiment, the plants were clipped off and various morphological parameters such as plant height, plant diameter, and plant fresh and dry weight were measured. Plant height was measured from the shoot tips to the root hairs with the help of measuring tape. Plant diameter was measured with the help of Vernier caliper (ST22302 SG Tools, Hangzhou, China). Total fresh weight was determined by measuring the weight of roots and shoots using weighting balance and plant samples were oven-dried for 72 h at 65 °C and thereafter plant dry weight was measured until the dry weight became constant. These dried samples were grounded into powdered form in a stainless-steel mortar and pestle for further analysis. The leaves were also collected for assessing enzymatic activity, washed with distilled water, and placed in liquid nitrogen and stored in a freezer at low temperature (−80 °C) for further analysis [44].

### 2.3. Determination of Chlorophyll Contents and Gaseous Exchange Parameters

For chlorophyll content analysis, 0.1 g of fresh leaf sample was extracted with 8 mL of 95% acetone for 24 h at 4 °C in the dark. The absorbance was measured by a spectrophotometer (UV-2550; Shimadzu, Kyoto, Japan) at 646.6, 663.6, and 450 nm. Chlorophyll content was determined by the standard method of Arnon [45].

At the same days, gaseous exchange was also measured. Net photosynthesis (*Pn*), leaf stomatal conductance (*gs*), transpiration rate (*Ts*), and intercellular carbon dioxide concentration (*Ci*) were measured from three different plants in each treatment group. Measurements were conducted between 11:30 and 13:30 on days with clear sky. Rates of leaf *Pn*, *gs, Ts*, and *Ci* were measured with a LI-COR gas-exchange system (LI-6400; LI-COR Biosciences, Lincoln, NE, USA) with a red-blue LED light source on the leaf chamber. In the LI-COR cuvette, CO_2_ concentration was set as 380 mmol mol^−1^ and LED light intensity was set at 1000 mmol m^−2^ s^−1^, which is the average saturation intensity for photosynthesis in *C. capsularis* [46].

### 2.4. Determination of Contents of Malondialdehyde and Proline and Activities of Antioxidant Enzyme

The degree of lipid peroxidation was evaluated as malondialdehyde (MDA) content. Briefly, 0.1 g of frozen leaves were ground at 4 °C in a mortar with 25 mL of 50 mM phosphate buffer solution (pH 7.8) containing 1% polyethylene pyrrole. The homogenate was centrifuged at 10,000× *g* at 4 °C for 15 min. The mixtures were heated at 100 °C for 15–30 min and then quickly cooled in an ice bath. The absorbance of the supernatant was recorded by using a spectrophotometer (xMark™ microplate absorbance spectrophotometer; Bio-Rad, USA) at wavelengths of 532, 600, and 450 nm. Lipid peroxidation was expressed as l mol g^−1^ using the following formula: 6.45 (A532-A600)-0.56 A450. Lipid peroxidation was measured using a method previously published by Health and Packer [47].

Proline contents were determined by using (0.1 g) homogenate in 3% of aqueous sulphosalicylic acid and distilled water. The proline content was assessed by the technique described by Bates et al. [48].

To evaluate enzyme activities, fresh leaves (0.5 g) were homogenised in liquid nitrogen and 5 mL of 50 mmol sodium phosphate buffer (pH 7.0) including 0.5 mmol EDTA and 0.15 mol NaCl. The homogenate was centrifuged at 12,000× *g* for 10 min at 4 °C, and the supernatant was used for measurement of SOD and POD activities. SOD activity was assayed in 3 mL reaction mixture containing 50 mM sodium phosphate buffer (pH 7), 56 mM nitroblue tetrazolium, 1.17 mM ribolavin, 10 mM methionine, and 100 μL enzyme extract. Finally, the sample was measured by using spectrophotometer (xMark™ microplate absorbance spectrophotometer; Bio-Rad). Enzyme activity was measured using a method by Chen and Pan [49], and expressed as U g^−1^ FW.

POD activity in the leaves was estimated using the method of Sakharov and Ardila [49] using guaiacol as the substrate. A reaction mixture (3 mL) containing 0.05 mL of enzyme extract, 2.75 mL of 50 mM phosphate buffer (pH 7.0), 0.1 mL of 1% H_2_O_2_, and 0.1 mL of 4% guaiacol solution was prepared. Increases in the absorbance at 470 nm due to guaiacol oxidation were recorded for 2 min. One unit of enzyme activity was defined as the amount of the enzyme.

### 2.5. Cu Determination

Dried root and shoot (leaves and stems) samples were ground in a stainless-steel mill and passed through a 0.1-mm nylon sieve for Cu analysis. Briefly, 0.1 g of dried sample was digested in HNO_3_/HClO_4_ (4:1) solution. Digested solution was washed in 25-mL flasks and diluted in de-ionised water until reaching the final volume of 25-mL. The supernatant was passed through a 0.45-μm filter paper and analysed for Cu content by an atomic absorption spectrophotometer (240FS-AA; Agilent).

Bioaccumulation factor (BAF) was calculated as the ratio of Cu content in tissues and Cu content in nutrient medium by using the following formula:

(1) $$\mathrm{BAF}=\frac{{\mathrm{Cu}\;\mathrm{contents}}_{\left(\mathrm{plant}\;\mathrm{tissues}\right)}}{{\mathrm{Cu}\;\mathrm{contents}}_{\left(\mathrm{nutrient}\;\mathrm{media}\right)}}$$

while translocation factor (TF) was determined by estimating the concentration of Cu in one part of plant with respect to the other parts as follow:

(2) $$\mathrm{TF}=\frac{{\mathrm{Cu}\;\mathrm{contents}}_{\left(\mathrm{shoots}\right)}}{{\mathrm{Cu}\;\mathrm{contents}}_{\left(\mathrm{roots}\right)}}$$

### 2.6. Transmission Electron Microscopy

Small sections of the leaves (1–3 mm in length) were fixed in 4% glutaraldehyde (*v*/*v*) in 0.2 mol/L SPB (sodium phosphate buffer, pH 7.2) for 6–8 h and post-fixed in 1% OsO_4_ for 1 h, then in 0.2 mol/L SPB (pH 7.2) for 1–2 h. Samples were dehydrated in a graded ethanol series (50%, 60%, 70%, 80%, 90%, 95%, and 100%) followed by acetone, filtered, and embedded in Spurr’s resin. Ultra-thin sections (80 nm) were prepared and mounted on copper grids for observation under a transmission electron microscope (JEOL TEM-1200EX) at an accelerating voltage of 60.0 kV or 80.0 kV.

### 2.7. Statistical Analysis

All values reported in this experiment are mean of three independent replicates mean ± SD. All the data obtained was tested by one-way ANOVA. Thus, the differences between treatments were determined using analysis of variance, and the least significant difference test (*p* < 0.05) used for multiple comparisons between treatment means. Pearson’s correlation analysis was performed to quantify relationships between various analyzed variables. The data recorded were statistically analyzed using Statistix 8.1 (Analytical Software, Tallahassee, FL, USA). The graphical representation was performed using SigmaPlot-10 and RStudio.

## 3. Results

### 3.1. Plant Growth and Biomass

Growth in terms of plant height, plant diameter, fresh and dry biomass were significantly (*p* < 0.05) decreased with the exposure of high Cu levels (50 and 100 μM) without the application of CA compared with control (Table 1). The maximum plant height, plant diameter, fresh weight, and dry weight reductions were recorded at highest Cu treatment i.e., 100 μM which caused 37.3%, 19.9%, 34.8%, and 33.1% reduction, respectively, as compared to control. The application of CA in the nutrient solution of Cu contaminated mixture with *C. capsularis* significantly (*p* < 0.05) revoked metal toxicity and increased plant growth and biomass. The results revealed that in Cu stressed plants (i.e., 100 μM) with the application of CA i.e., 2 mM exhibited 40.3%, 18.1%, 40.8%, and 33.3% increase in plant height, plant diameter, fresh weight, and dry weight, respectively, when compared to 100 μM Cu without the application of CA.

### 3.2. Chlorophyll Contents and Gaseous Exchange Attributes

Results regarding different levels of Cu (0, 50, and 100 μM) with or without the application of CA on total chlorophyll and carotenoid contents of *C. capsularis* are presented in Table 1. These results revealed that when *C. capsularis* was grown under different levels of Cu i.e., 50 and 100 μM significantly (*p* < 0.05) reduced total chlorophyll and carotenoid contents as compared to control. However, application of CA to the Cu-stressed plants significantly (*p* < 0.05) increased total chlorophyll and carotenoid contents in *C. capsularis*. The increase in total chlorophyll and carotenoid contents was 43.8% and 9.5% in 100 μM Cu stress level with the application of CA compared with the respective treatment without application of CA.

**Figure 1:**
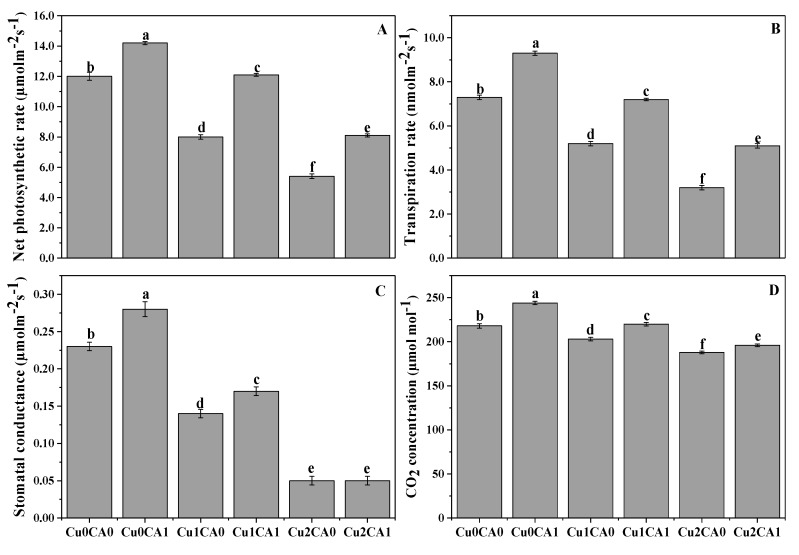
Effect of CA application on net photosynthesis (**A**), transpiration rate (**B**), stomatal conductance (**C**) and intercellular CO_2_ concentration (**D**) in the leaves of *C. capsularis* seedlings grown under different stress levels of Cu. Values are demonstrated as means of three replicates along with standard deviation (SD; n = 3). One-way ANOVA was performed and means differences were tested by LSD (*p* < 0.05). Different lowercase letters on the error bars indicate significant difference between the treatments. Cu_0_CA_0_ (Cu = 0 μM and CA = 0 mM), Cu_0_CA_1_ (Cu = 0 μM and CA = 2 mM), Cu_1_CA_0_ (Cu = 50 μM and CA = 0 mM), Cu_1_CA_1_ (Cu = 50 μM and CA = 2 mM), Cu_2_CA_0_ (Cu = 100 μM and CA = 0 mM), Cu_2_CA_1_ (Cu = 100 μM and CA = 2 mM).

Cu stress (50, 100 μM) significant (*p* < 0.05) reduced net photosynthetic rate (P*n*), transpiration rate (T*r*), stomatal conductance (G*s*) and intercellular CO_2_ (C*i*) (Figure 1). These results showing that the application of CA to Cu treated plants significant increased gaseous exchange attributes compared with the plants grown under Cu-only treatment. The application of CA to the plants treated with 50 μM cause 51.2%, 38.4%, 21.4%, and 8.4% increase in P*n*, T*r*, G*s*, and C*i*, respectively, when compared with the plants grown under 50 μM without the application of CA (Figure 1A–D). In the same way, the plants grown under 100 μM Cu treated with CA significantly increase P*n*, T*r*, G*s*, and C*i* by 50%, 59.3%, 20%, and 4.2%, respectively, compared with the plants grown under 100 μM without the application of CA (Figure 1A–D).

### 3.3. Oxidative Stress and Antioxidant Enzyme Activities

**Figure 2:**
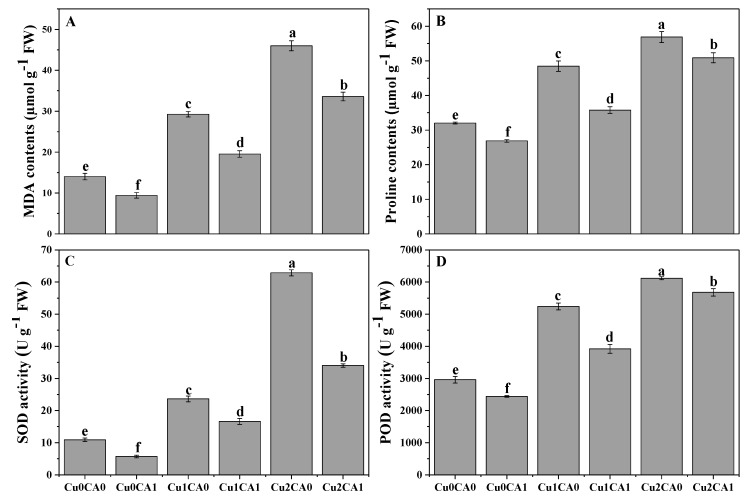
Effect of CA application on Cu-induced malondialdehyde (MDA) (**A**), proline contents (**B**), and antioxidative defense mechanisms such as activities of superoxide dismutase (SOD) (**C**) and peroxidase (POD) (**D**) in the leaves of *C. capsularis* seedlings grown under different stress levels of Cu. Values are demonstrated as means of three replicates along with standard deviation (SD; n =3). One-way ANOVA was performed and means differences were tested by LSD (*p* < 0.05). Different lowercase letters on the error bars indicate significant difference between the treatments. Cu_0_CA_0_ (Cu = 0 μM and CA = 0 mM), Cu_0_CA_1_ (Cu = 0 μM and CA = 2 mM), Cu_1_CA_0_ (Cu = 50 μM and CA = 0 mM), Cu_1_CA_1_ (Cu = 50 μM and CA = 2 mM), Cu_2_CA_0_ (Cu = 100 μM and CA = 0 mM), Cu_2_CA_1_ (Cu = 100 μM and CA = 2 mM).

It was noticed that increasing levels of Cu concentration in the nutrient solution caused increasing contents of malondialdehyde (MDA) contents in the leaves of *C. capsularis* (Figure 2A). Furthermore, increasing contents of MDA contents proposed that Cu toxicity induced oxidative damage in the leaves of *C. capsularis* (Figure 2A). However, the application of CA decreased MDA contents and reduced oxidative damage in the leaves of *C. capsularis*. According to the results, increasing levels of Cu (50 and 100 μM) in the nutrient solution increased MDA contents by 108.5% and 228.5%, respectively, and increased proline contents by 51.2% and 77.5%, respectively, compared to the treatment without Cu and CA. Results also showing that addition of CA caused a significant decreased in MDA and proline contents and decreased by 27.1% and 10.7%, respectively, at 100 μM with the application of CA, compared to the plants grown under 100 μM without the application of CA.

Results related to antioxidative activities of enzymes (SOD and POD) in the leaves of *C. capsularis* are presented in (Figure 2C, D). It was observed that increasing level of Cu in the nutrient solution significantly (*p* < 0.05) increased the enzymatic activity of SOD and POD in the leaves of *C. capsularis* but the application of CA significant decrease the activity of antioxidants compared with the plants grown under without application of CA. The maximum increased in the activities of SOD and POD were observed at Cu level of 100 μM which was increased by 476.1% and 106.8% compared to the control treatment (without addition of Cu and CA in the nutrient solution). However, application of CA decreased the activities of SOD and POD in both Cu levels (50 and 100 μM).

### 3.4. Uptake and Distribution of Cu

**Figure 3:**
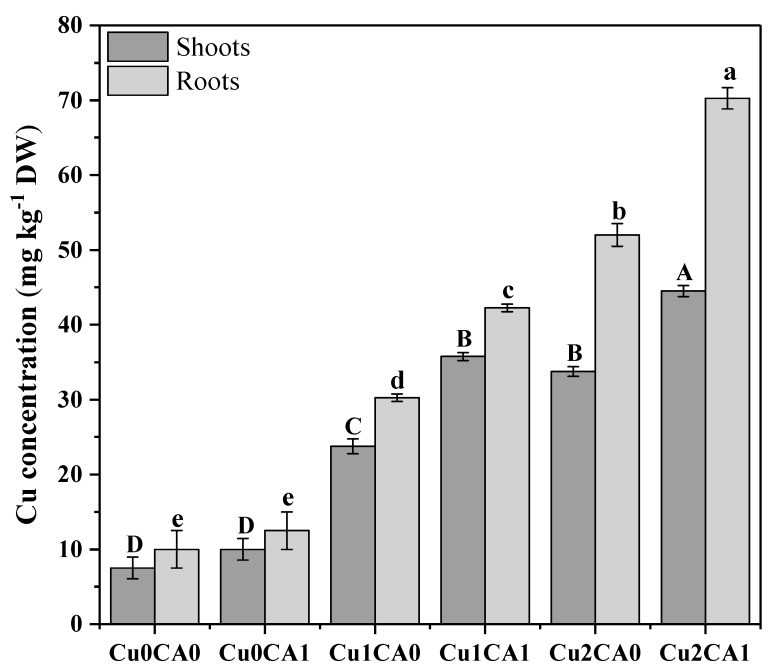
Cu uptake and accumulation by different plant parts (shoots and roots tissues) of *C. capsularis* seedlings under different levels of Cu in the nutrient solution with the exogenous supplementation of CA. Values are demonstrated as means of three replicates along with standard deviation (SD; n = 3). One-way ANOVA was performed and means differences were tested by LSD (*p* < 0.05). Different uppercase and lowercase letters on the error bars indicate significant differences between the treatments within shoots and roots, respectively. Cu_0_CA_0_ (Cu = 0 μM and CA = 0 mM), Cu_0_CA_1_ (Cu = 0 μM and CA = 2 mM), Cu_1_CA_0_ (Cu = 50 μM and CA = 0 mM), Cu_1_CA_1_ (Cu = 50 μM and CA = 2 mM), Cu_2_CA_0_ (Cu = 100 μM and CA = 0 mM), Cu_2_CA_1_ (Cu = 100 μM and CA = 2 mM).

The results regarding Cu uptake showed that the increasing concentration of Cu in nutrient solution significantly (*p* < 0.05) increased Cu concentration in the roots and shoots of *C. capsularis* (Figure 3). It was also noticed that application of CA to the Cu stressed plants also helps in the Cu accumulation in *C. capsularis*. It was noticed that highest concentration of Cu was observed in roots (70 mg kg^−1^) followed by shoots (46 mg kg^−1^) of *C. capsularis* at 100 μM with the application of CA in the nutrient solution. Among different treatments the highest concentration of Cu in the roots was recorded at 100 μM with the application of CA (70 mg kg^−1^) followed by 100 μM without the application of CA (52 mg kg^−1^) and 50 μM with the application of CA (42 mg kg^−1^). These results suggested that higher Cu in the nutrient solution cause high concentration of Cu in the roots and shoots of *C. capsularis*. However, application of CA significantly increased Cu concentration in the roots and shoots of *C. capsularis*. Maximum concentration of Cu in the leaves was observed at 100 μM with the application of CA (46 mg kg^−1^) followed by 100 μM without the application of CA (36 mg kg^−1^) and 50 μM with the application of CA (36 mg kg^−1^). Application of CA increases total Cu concentration in roots and leaves as compared to the Cu treated plants without CA.

Bioaccumulation factor (BAF) and translocation factor (TF) in *C. capsularis* seedlings are shown in (Table 2). It was noticed that the values of BAF and TF were less in plants without application of CA, however, application of CA to Cu stressed plants showed higher values of BAF and TF as compare to the plants without CA. The minimum value of TF was observed in 100 μM with the application of CA (0.64) while maximum TF value was observed in 50 μM with the application of CA (0.83) (Table 2). The highest BAF value was recorded at 50 μM with the application of CA (0.84) in the roots while (0.70) in the shoots (Table 2). Cu uptake was highest in the roots than shoots while application of CA increases the value of BAF and TF.

### 3.5. Transmission Electron Microscopy

**Figure 4:**
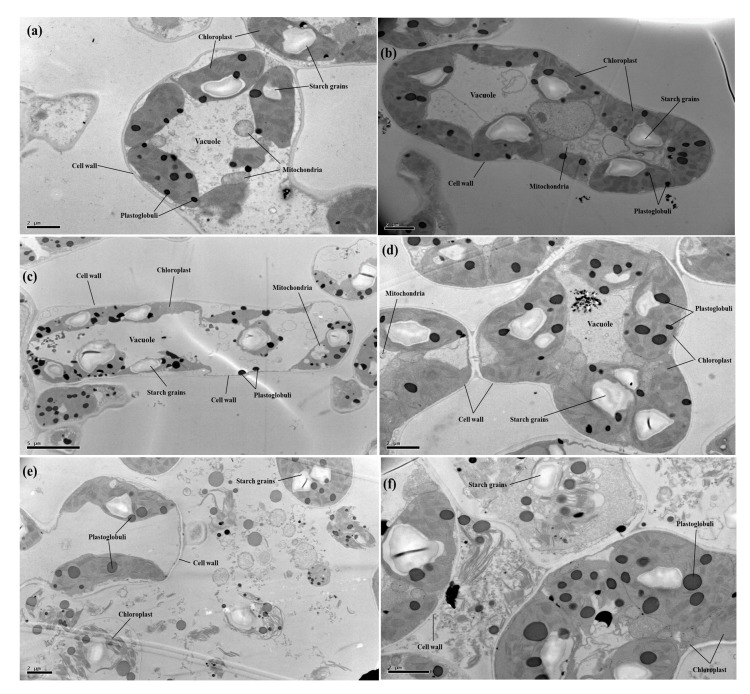
Transmission electron microscopy (TEM) images of *C. capsularis* leaf cells. The abbreviations are as follows: (**a**) Cu_0_CA_0_ (10,000), (**b**) Cu_0_CA_1_ (10,000), (**c**) Cu_1_CA_0_ (5000), (**d**) Cu_1_CA_1_ (10,000), (**e**) Cu_2_CA_0_ (5000), and (**f**) Cu_2_CA_1_ (10,000).

In the present study, effects of different levels of Cu (0, 50, and 100 μM) with or without the application of CA (2 mM) on cellular structure of *C. capsularis* seedlings were also observed under transmission electron microscopy (TEM) (Figure 4). TEM results showed ultra-structural alterations in many cellular organelles especially cellular bounded organelles of the *C. capsularis* leaf cell. At 50 μM Cu, the Cu distribution percent in organelles in the root and leaf cells decreased, but that in the cell wall and soluble fraction increased, as compared to that at 0 μM Cu. The decrease in Cu distribution percent observed at exposure concentrations that affect the structure of many organelles may just be due to effects on transport systems. For the same Cu exposure level and exposure time, Cu is mainly deposited in the cell wall, then chloroplast and the soluble fraction in the plant leaf cells. After increasing Cu to 100 μM, the distribution percent of Cu in the cell wall and chloroplast was increased distribution percentage in the cell wall and decreased distribution percentage in the chloroplast were noted in the plant leaf cells. In those of organelles, chloroplast structure appeared degenerated, and even broken (completely dispersed), leaving plastoglobuli and starch grains and other cellular organelles with the cytoplasm. However, compared to the Cu treatments, it was also revealed that application of CA improved cellular organelles, especially chloroplast structure of the *C. capsularis* leaf cell (Figure 4).

### 3.6. Correlation Analysis

**Figure 5:**
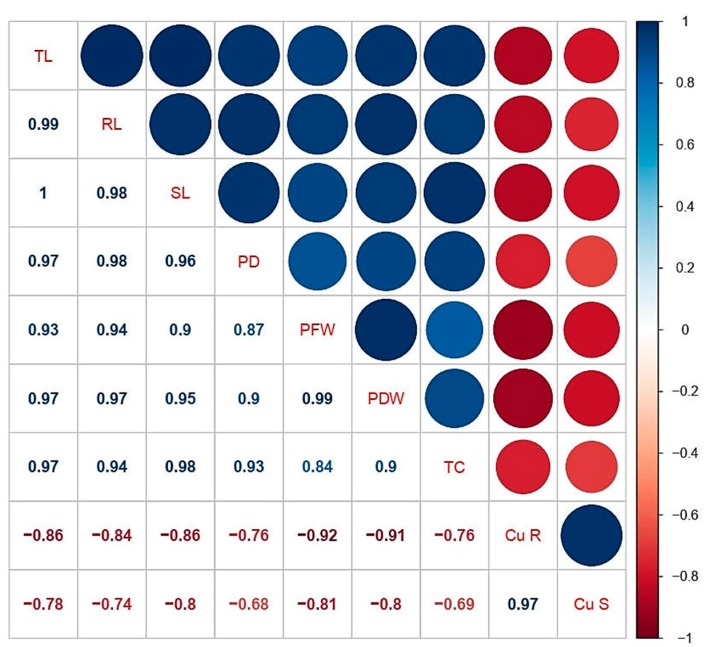
Relationship (r values) between different studies parameters of *C. capsularis* seedlings grown under different stress levels of Cu with and without CA application. The abbreviations are as follows: TL: total plant length; RL: root length; SL: shoot length; PD: total plant diameter; PFW: plant fresh weight; PDW: plant dry weight; TC: total chlorophyll contents; Cu. S: Cu in shoots; and Cu. R: Cu in roots.

A Pearson’s correlation analysis was executed between different studied parameters of *C. capsularis* are shown in (Figure 5). According to the correlation analysis it was noticed that Cu concentrations in the roots was positively correlated with Cu concentration in the shoots while negatively correlated with plant height, plant fresh weight, plant diameter, plant fresh weight, plant dry weight, chlorophyll, and carotenoid contents. Similarly, Cu concentration in the shoots was positively correlated with Cu concentration in the roots while negatively correlated with other morphological and physiological traits. This correlation depicts a close connection between Cu uptake and other parameters studied of *C. capsularis*.

### 3.7. Principal Component Analysis

**Figure 6:**
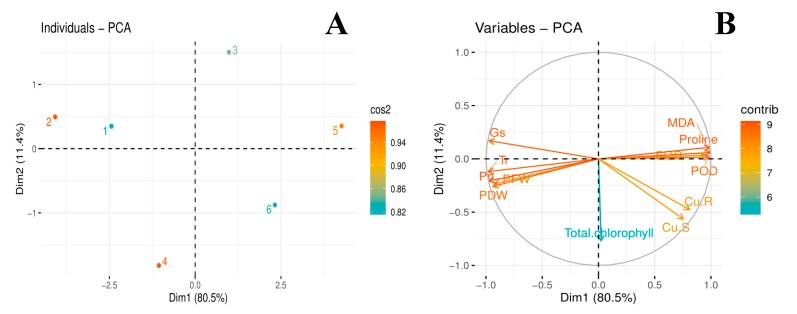
Score (**A**) and loading plot (**B**) of principal component analysis (PCA) on different studied attributes of *C. capsularis* seedlings plants supplemented with citric acid (CA) while grown under Cu stress. Score plot represents separation of treatments as (1) Cu_0_CA_0_ (Cu = 0 μM and CA = 0 mM), (2) Cu_0_CA_1_ (Cu = 0 μM and CA = 2 mM), (3) Cu_1_CA_0_ (Cu = 50 μM and CA = 0 mM), (4) Cu_1_CA_1_ (Cu = 50 μM and CA = 2 mM), (5) Cu_2_CA_0_ (Cu = 100 μM and CA = 0 mM) and (6) Cu_2_CA_1_ (Cu = 100 μM and CA = 2 mM). The abbreviations are as follows: PFW: plant fresh weight; PDW: plant dry weight; MDA: Lipid peroxidation; Pn: photosynthetic rate; Tr: transpiration rate; Gs; stomatal conductance; POD: peroxidase activity; SOD: superoxidase dismutase activity; Cu. S: Cu in shoots; and Cu. R: Cu in roots.

The score and loading plots of principal component analysis (PCA) to evaluate Cu and citric acid treatment effects on some important studied attributes of jute (C. capsularis) plants are presented in (Figure 6). Among all the components, first two components i.e., PC1 (Dim1) and PC2 (Dim2) exhibited maximum contribution and accounted for 91.9% of the total variance in the dataset. Of which, PC1 contributed 80.5%, while PC2 contributed 11.4%, accordingly. All of the 6 treatments were dispersed successfully by first two principal components (Figure 6A). This distribution of treatments gave a clear indication that citric acid application under Cu stress had a significant ameliorative effect on studied attributes of jute plants compared to control. The Cu treatments without citric acid i.e., 50 µM (3) and 100 μM (5) were more displaced from the treatments such as, control (1); citric acid amendment under 50 μM Cu stress (4) and citric acid amendment under 100 μM Cu stress (6) (Figure 6A), indicating that Cu-stress imposed hazardous impacts on growth and ecophysiology of jute plants. The first group of variables with which PC1 is positively correlated includes the variables such as POD; SOD; Proline; MDA; Cu-R; and Cu-S. Contrarily, a significant negative correlation of PC1 variables was found with the variables aligned with PC2: PFW; PDW; Total chlorophyll; Pn; Tr; and Gs (Figure 6B).

## 4. Discussion

Excess of Cu is highly toxic for normal growth of plants. Reduction in plant growth and biomass is a common response in plants exposed to an excess of Cu [7,50]. In the present study, increasing concentration of Cu (50 and 100 μM) in the nutrient solution significantly decreased plant height, plant diameter, total fresh and dry weight compared to the control treatment (Table 1). The reduction in plant growth and biomass might be related with disturbed metabolic activities because of decreased take-up of fundamental mineral nutrients when developed under Cu toxicity [1,8,51]. Moreover, plants species which can generate high shoot (above-ground) biomass and have the capacity to accumulate heavy metals could be utilized for phytoextraction purposes including exclusion of heavy metals from contaminated soil [25,27]. Various examinations demonstrate the phytotoxic effects of increased levels of Cu on plant growth and biomass cultivated in Cu contaminated soil [6,30,52]. In our previous study on kenaf (*Hibiscus cannabinus* L.) seedlings, when exposed to short-term (14 days) exposure of Cu-stress, we concluded that increasing concentration of Cu (60, 120, and 180 μM) in the nutrient solution, significantly decreased plant height, plant diameter, total fresh and dry biomass of the plant compared to the control treatment [16]. Furthermore, our outcomes for Cu phytotoxicity were obvious from hindered growth and development and also reduced fresh and dry weights that are in consonance with a similar studies on ramie, maize, and falx seedlings under Cu stress [5,21,53].

Additionally, Cu stress induced a negative impact on leaf chlorophyll contents and gas exchange attributes. This might be the consequence of disruption of chloroplast, protein complex, and photosynthetic apparatus when plants are exposed to heavy metal stress [5,42,54]. The main effects of Cu on photosynthesis are related to changes in pigment compositions and ultrastructure of chloroplast, decreased net photosynthesis rate, reduced ribulose-1,5-bisphosphate carboxylase/oxygenase (RuBisCo) efficiency, and inhibition of electron transport and PSII activities [3,30]. Another possible reason for decreased gaseous exchange attributes might be the replacement of Mg^2+^ ions by Cu which is an important element for chlorophyll biosynthesis and ultra-structure of chloroplast which is disrupted by metal toxicity [8,55]. Uptake of Cu into above ground parts of C. capsularis damages the photosynthetic machinery via impaired lamellar membrane of chloroplast. These alterations in chloroplast cause manipulation of photosynthesis, leading to stunted plant growth [1,4].

Cu in excess causes generation of reactive oxygen species (ROS) such as superoxide radical (O^.-^), H_2_O_2_, singlet oxygen (^1^O_2_), and hydroxyl radicals (OH) [6,11,53,56]. Plants have a variety of antioxidants such as superoxide dismutase (SOD) and peroxidase (POD) and some specific metabolites play a key role in adaptation and survival of plants under metal toxicity. Oxidative stress alters the antioxidant activities, which are essential to mitigate this stress [12,57]. It was reported that excess of Cu can increase lipid peroxidation [22,58] and MDA, an oxidized product of membrane lipids, indicating the prevalence of oxidative stress and membrane damage [5]. Moreover, when plants exposed to excess Cu have been shown to accumulate proline in their tissues [9]. In our study, Cu toxicity increased oxidative stress in the leaves of *C. capsularis* while antioxidant enzymes come into play to reduce metal toxicity (Figure 2). SOD and POD activities have been studied in Cu-stressed rapeseed, rice and jute plants [22,30,36]. Up-regulation of activity of various antioxidative enzymes shows the capacity of plants to scavenge excessive ROS in the cells. The enhanced activities of antioxidant enzymes might be due to the increased level of ROS [6,30,52]. Plants try to survive under stressful environments, by activating enzymatic defense system, which scavenges ROS by regulating the K^+^ efflux and electron transport chain [11,53,58].

The translocation factor (TF) and bioaccumulation factor (BAF) are important in screening hyperaccumulators for phytoremediation of heavy metals. Screening of hyperaccumulators depend on BAF and TF values (both of them are greater than 1) for evaluation and selection of plants for phytoremediation [7,21]. The TF is the capacity of plants to transfer metals from roots to shoots and BAF express the ability of plants to accumulate metals from soils to tissues [59]. In the present study, most of the Cu was accumulated in the roots while a little was transported to the shoots (Figure 3). A hyper accumulator plants had the ability to absorb and accumulate heavy metals in high concentrations in their above ground tissues without severe damage to vital physiological processes and plant growth [59]. In a pot experiment, we measured BAF and TF values of *C. capsularis* at different stages of growth of *C. capsularis* plants [36]. However, in that study, we noticed that at earlier stage of the growth Cu was highly accumulated in the roots while at the lateral stages of the growth Cu was highly transported to the shoots (aboveground parts of the plants) due to the formation of iron-plague in the roots. In this study, we designed the experiment just for 21 days to conclude our results. Hence, Cu was highly accumulated in the roots and all the values of BAF and TF are less than 1 which we also concluded in a petri dish experiment with short-term exposure of Cu stress, that Cu was mainly accumulated in the roots while a little transported to the shoots [3]. Previously many studies reported that *C. capsularis* is a hyperaccumulator species for different heavy metal [60,61,62].

The chloroplasts, nucleus, mitochondrion, and ribosomes are the key cell organs in the plant cell, for the major cell life activities. Cu excess may also result in membrane damage by Cu binding to the sulfhydryl groups of membrane proteins [2]. At proper levels, Cu can keep the structure steady in the organelle membrane while at excessive levels, it can damage the integrity of the membrane structure within the plant cell [43,63]. Although, chloroplast is the main site of Cu accumulation as mentioned in the details in the review of literature by Adrees et al. [2]. In the present study, increasing Cu level in the nutrient solution disturbs the ultra-structure of chloroplast (Figure 4) and maximum Cu level (100 μM) caused a severe damage to all membrane bounded organelles (Figure 4). Although, there is no previous study of *C. capsularis* to study ultra-structure alteration of chloroplast under Cu-stress, but we demonstrated in pot experiment that Cu toxicity disturbs cellular organelles in *C. capsularis* plants while fertilization of P improved membrane bounded structures which were investigated with TEM analysis [18]. However, similar results we noticed in another pot experiment that Cu toxicity disrupts the ultra-structure of chloroplast [37].

The promotive role of CA in plants exposed to heavy metal stress is well recognized. Recent studies have documented the role of CA as a growth promoting agent with a chelating potential against different heavy metals such as Cu [30], Cr [39], Pb [64], as well as Cd [65]. The application of CA improved the growth and biomass of *C. capsularis* seedlings under Cu stress (Table 1). Results of the present study were in line with the outcome described by Farid et al. [39], Najeeb et al. [43], Niazy and Wahdan [64], and Shakoor et al. [42]. Although the application of CA is independent to metal stress as it increased plant growth and biomass (even under normal condition), this might be due to increased nutrient uptake and/or CA induced chelation of metals decreasing free metal ions in plants as suggested by Zaheer et al. [30]. Moreover, the CA application also increased photosynthetic pigments, gaseous exchange attributes, and ultrastructure of chloroplast which is linked with the improvement in plant growth and biomass as suggested by Zaheer et al. [30]. As suggested by Mallhi et al. [66], improvement in plant growth and biomass under heavy metal stress condition might be due to the chelating role of CA, which helps to increase nutrient uptake by the plant. Improvement in plant growth and biomass might be accredited to the ability of CA to enhance the uptake of essential nutrients by the formation of complexes with nutrients [67]. The other possible reason might be that the application of CA may enhance the photosynthesis and synthesis of phytochelatins (PCs) in plants [64,68]. In the present study, application of CA predominantly reduced the generation of ROS and proline contents and also decreased the activities of antioxidative enzymes compared to Cu-stressed plants (Figure 2). Antioxidant defensive activities play a novel role regarding the reduction of ROS production. In numerous studies application of chelating agents increased the activities of antioxidative enzymes and reduced oxidative stress by decreased the generation of ROS production [30,54,68]. However, sometimes the activities of antioxidant showed duel behavior, may be increased its activities with the application of chelating agents or sometimes it may be decreased under heavy metal stress environment [66]. This might be due to the growth promotive character of CA in assisting the plant to recover fast from oxidative damage [69].

Our results also showed that application of CA significantly increased Cu uptake in different parts of plant body (Figure 1). In many previous studies it was observed that application of CA is helpful to increase phytoextraction of heavy metals using different plant species such as *Brassica napus* [30], *Brassica juncea* [65], and Zea mays [41]. Increase in Cu uptake by plants might be due to chelation of Cu with CA [30]. CA application also increased plant growth and biomass and, consequently, the accumulation and uptake of metals in plants. This relative increase in Cu contents might be due to CA-induced increase transpiration rate, which in turn increased Cu translocation to shoot through water movement and/or due to Cu chelation [43,70].

## 5. Conclusions

On the basis of these results, it can be concluded that Cu toxicity reduced plant height, plant diameter, plant fresh and dry weight, total chlorophyll and carotenoid contents, gaseous exchange attributes, and affected chloroplast structure while scavenging ROS production. However, Cu uptake and accumulation increases in *C. capsularis* by increased Cu concentration in the nutrient solution. The negative impact of excess Cu can overcome with the application of CA which significantly increased plant growth and biomass, photosynthetic pigments, gaseous exchange attributes, and improved cellular organelles of the plant cell and reduce the oxidative stress by lowering MDA and proline contents and thereby normalized the enzymatic activities of SOD and POD. Moreover, application of CA also increases the Cu uptake in the *C. capsularis*, thus helps in phytoextraction of Cu efficiently. Conclusively, the obtained results suggested that the application of CA increased Cu accumulation by reducing its toxicity and thereby improved the growth and biomass of *C. capsularis* in the presence as well as the absence of Cu treatment. Therefore, *C. capsularis* can be used as phytoextraction of heavy metals such as Cu with the application of CA but further soil-based studies are required to validate these results.

## Figures and Tables

**Table 1:** Effect of citric acid (CA) application on plant growth, biomass and photosynthetic pigments of *C. capsularis* seedlings grown under different stress levels of Cu.

Treatments	Plant Height (cm)	Plant Diameter (mm)	Plant Fresh Weight (g)	Plant Dry Weight (g)	Total Chlorophyll (mg g^−1^ FW)	Carotenoids (mg g^−1^ FW)
Cu_0_CA_0_	19.8 ± 0.18 c	2.06 ± 0.02 c	2.18 ± 0.03 c	1.48 ± 0.02 b	2.8 ± 0.04 b	1.01 ± 0.02 b
Cu_0_CA_1_	24.6 ± 0.40 a	2.45 ± 0.04 a	2.59 ± 0.04 a	1.73 ± 0.03 a	3.2 ± 0.03 a	1.08 ± 0.01 a
Cu_1_CA_0_	14.8 ± 0.35 e	1.84 ± 0.02 e	1.91 ± 0.03 e	1.23 ± 0.02 d	1.99 ± 0.04 d	0.82 ± 0.04 d
Cu_1_CA_1_	20.7 ± 0.25 bc	2.15 ± 0.03 b	2.31 ± 0.02 b	1.54 ± 0.02 b	2.7 ± 0.04 c	0.89 ± 0.02 c
Cu_2_CA_0_	12.4 ± 0.35 f	1.65 ± 0.02 f	1.42 ± 0.01 f	0.99 ± 0.03 e	1.6 ± 0.03 f	0.74 ± 0.01 e
Cu_2_CA_1_	17.4 ± 0.35 d	1.95 ± 0.04 d	2 ± 0.05 de	1.32 ± 0.03 c	2.3 ± 0.06 e	0.81 ± 0.03 d

The given values are means ± SD (n =3). One-way ANOVA was performed and means differences were tested by least significant difference LSD (*P* < 0.05). Different lowercase letters in the table indicate significant difference between the treatments. Cu_0_CA_0_ (Cu = 0 μM and CA = 0 mM), Cu_0_CA_1_ (Cu = 0 μM and CA = 2 mM), Cu_1_CA_0_ (Cu = 50 μM and CA = 0 mM), Cu_1_CA_1_ (Cu = 50 μM and CA = 2 mM), Cu_2_CA_0_ (Cu = 100 μM and CA = 0 mM), Cu_2_CA_1_ (Cu = 100 μM and CA = 2 mM).

**Table 2:** Effect of citric acid (CA) application on bioaccumulation factor (BAF) and translocation factor (TF) of *C. capsularis* seedlings grown under different stress levels of Cu.

Treatments	BAF (Roots)	BAF (Shoots)	TF
Cu_1_CA_0_	0.60	0.46	0.76
Cu_1_CA_1_	0.84	0.70	0.83
Cu_2_CA_0_	0.52	0.33	0.65
Cu_2_CA_1_	0.70	0.44	0.64

Cu_1_CA_0_ (Cu = 50 μM and CA = 0 mM), Cu_1_CA_1_ (Cu = 50 μM and CA = 2 mM), Cu_2_CA_0_ (Cu = 100 μM and CA = 0 mM), Cu_2_CA_1_ (Cu = 100 μM and CA = 2 mM).
